# The Long Pentraxin PTX3 Is of Major Importance Among Acute Phase Proteins in Chickens

**DOI:** 10.3389/fimmu.2019.00124

**Published:** 2019-02-01

**Authors:** Nina B. Burkhardt, Susanne Röll, Anke Staudt, Daniel Elleder, Sonja Härtle, Taiana Costa, Andreas Alber, Mark P. Stevens, Lonneke Vervelde, Benjamin Schusser, Bernd Kaspers

**Affiliations:** ^1^Department for Veterinary Sciences, Ludwig-Maximilians-Universität Munich, Munich, Germany; ^2^Institute of Molecular Genetics of the Academy of Sciences of the Czech Republic, Prague, Czechia; ^3^The Roslin Institute and Royal (Dick) School of Veterinary Studies, University of Edinburgh, Edinburgh, United Kingdom; ^4^Reproductive Biotechnology, Technical University of Munich, School of Life Sciences Weihenstephan, Freising, Germany

**Keywords:** chicken, pentraxin, acute phase proteins, inflammation, next generation sequencing, LPS, avian pathogenic *E. coli*

## Abstract

The expression level of acute phase proteins (APPs) mirrors the health status of an individual. In human medicine, C-reactive protein (CRP), and other members of the pentraxin family are of significant relevance for assessing disease severity and prognosis. In chickens, however, which represent the most common livestock species around the world, no such marker has yet gained general acceptance. The aim of this study was therefore, to characterize chicken pentraxin 3 (chPTX3) and to evaluate its applicability as a general marker for inflammatory conditions. The mammalian and chicken PTX3 proteins were predicted to be similar in sequence, domain organization and polymeric structure. Nevertheless, some characteristics like certain sequence sections, which have varied during the evolution of mammals, and species-specific glycosylation patterns, suggest distinct biological functions. ChPTX3 is constitutively expressed in various tissues but, interestingly, could not be found in splenic tissue samples without stimulation. However, upon treatment with lipopolysaccharide (LPS), PTX3 expression in chicken spleens increased to 95-fold within hours. A search for PTX3 reads in various publicly available RNA-seq data sets of chicken spleen and bursa of Fabricius also showed that PTX3 expression increases within days after experimental infection with viral and bacterial pathogens. An experimental infection with avian pathogenic *E.coli* and qPCR analysis of spleen samples further established a challenge dose-dependent significant up-regulation of chPTX3 in subclinically infected birds of up to over 150-fold as compared to untreated controls. Our results indicate the potential of chPTX3 as an APP marker to monitor inflammatory conditions in poultry flocks.

## Introduction

The poultry industry is a fast-growing and intensive branch of the food economy and it is likely to gain even more relevance in the future. This development is due to the chickens' extraordinarily good feed conversion ratio and their low methane and carbon dioxide production (1), which makes chickens a financially rewarding and environmentally sound meat source. To further maintain chicken health, parameters indicating pathological conditions before they are clinically visible are highly demanded, but no valuable marker for general inflammation in chickens has been established so far.

Potential candidates for such markers may be found within the class of the acute phase proteins (APPs) (2). APPs are positively or negatively regulated during the acute phase response (APR) as a reaction to proinflammatory cytokine secretion shortly after impact of inflammatory stimuli and represent an unspecific immune reaction (3, 4). They are classified as major (>10-fold), moderate (4 to 10-fold), or minor APPs (2 to 3-fold) according to their fold increase after application of an inflammatory stimulus (5). An increased expression of APPs, especially of members of the pentraxin family, has previously been associated with a variety of pathological conditions like cancer (6), acute and chronic inflammatory disease (7–9) and psychosocial stress (10) in mammals. C-reactive protein (CRP), which was discovered in 1930 (11), is among the most important markers for the severity of inflammatory diseases in clinical human medicine (12).

There is a growing body of information concerning the acute phase response in chickens. The most commonly examined chicken APPs are ceruloplasmin, ovotransferrin, and α1-acid glycoprotein (13–15). They are classified as moderate APPs (5). Ceruloplasmin and ovotransferrin are both important antioxidants. Ceruloplasmin catalyzes the oxidation of Fe^2+^ and thereby prevents the formation of free radicals (16). Ovotransferrin binds Fe^3+^ and its high affinity for this ion provides protection against iron-catalyzed free radical formation and also inhibits bacterial growth (17). α1-acid glycoprotein is a pH-dependent, unspecific carrier of lipophilic compounds, which is most effective in a basic or neutral environment (18). Surprisingly, most acute phase markers established in human medicine are classified only as minor or moderate APPs in chickens (5). CRP and mannose-binding lectin (MBL), for example, two of the major mammalian APPs, have been found to increase expression only around 2-fold in response to *Ascaridia galli* infestation (19). MBL is also weakly up-regulated in serum at 3 days post infection with IBV (20), 2 days after inoculation of E.coli (21), and 16 h after LPS challenge (22). However, a variable genetic background of chicken lines and individuals, that strongly influences the constitutive expression of MBL, has been found (23), indicating that the value of MBL—and therefore also of other minor and moderate APPs—as marker proteins must be considered carefully. The investigation of major chicken acute phase proteins hence is crucial for establishing a general health marker for this species.

SAA, which is in humans known to be up-regulated in osteoarthritis and might potentially contribute to the formation of the disease (24), has been investigated in chickens and is classified as the only major APP in this species so far (5). It was found that SAA serum concentrations increase strongly 24 to 48 h after *Enterococcus faecalis* injection (25) and other stimuli (26). More recent studies, however, revealed a less pronounced up-regulation of SAA after infection with IBDV and IBV (27, 28) and thereby object its classification as a major APP.

Among other APPs, such as haptoglobin, complement C3 and α2-macroglobulin, members of the pentraxin family are of particular relevance during the human APR (29–31). Pentraxin family members are characterized by a C-terminal pentraxin domain, which contains the eight amino acid pentraxin signature motif. This short sequence is highly conserved among pentraxins. While the so-called short pentraxins, like the classical APPs CRP and Serum amyloid P (SAP), consist solely of the pentraxin domain, the long family members like Pentraxin 3 (PTX3) possess a second, N-terminal domain (32). As the cysteine residues C47, C49, and C103 within the N-terminal domain of human PTX3 form interchain disulfide bridges (33), quaternary structures of the prototypic short and long pentraxins vary due to the missing N-terminal domain in short pentraxins (32). Human PTX3 forms asymmetric, cyclic octamers (33).

PTX3 has been studied intensively in mice and humans, revealing its multiple roles in immunity. It contributes to prevention of bacterial (34), viral (35), and fungal (36) infections and is associated with autoimmune diseases, such as systemic lupus erythematosus, ankylosing spondylitis and multiple sclerosis (37). Despite the magnitude of information concerning structure, function, and expression patterns of mammalian PTX3, the chicken equivalent has been neglected to date. Interestingly, our group recently found PTX3 to be strongly induced in chickens after IFN-α application. It became evident that, compared to control animals, PTX3 expression in spleens of birds that have been injected with this cytokine increased 685-fold 3 h after injection, whereas CRP was not detected at all (38). This massive increase in PTX3 expression after immune stimulation indicates the potential relevance of PTX3 among APPs in chickens.

In this study, we investigate the predicted protein structure of chPTX3 and its expression patterns under non-inflammatory and inflammatory conditions and thereby evaluate its importance as a general marker for inflammatory conditions in birds.

## Materials and Methods

### Sequence Alignment, Phylogenetic Analysis, and Prediction of Protein Characteristics

Species-specific PTX3 sequences were obtained from Ensemble (https://www.ensembl.org/; human: ENST00000295927.3; mouse: ENSMUST00000029421.5; bovine: ENSBTAT00000011863.4; porcine: ENSSSCT00000012837.4; chicken: ENSGALT00000045711.2; frog: ENSXETT00000026752.2; zebra fish: ENSDART00000112933.3). Sanger-sequencing of PCR products for alignment was commissioned to GATC Biotech AG, Konstanz, Germany. The sequences were aligned via ClustalX2 (http://www.clustal.org) and further edited with GeneDoc2.7 (http://genedoc.software.informer.com). Sequence similarities and identities were calculated using LALIGN by ExPasy (http://embnet.vital-it.ch/software/LALIGN_form.html). N-glycosylation sites were predicted using the NetNGlyc 1.0 Server (http://www.cbs.dtu.dk/services/NetNGlyc/). For phylogenetic analysis, protein sequences of the above mentioned species-specific PTX3 transcripts were obtained from ensemble and aligned via Muscle Alignment in MEGA (https://www.megasoftware.net/). The resulting alignment was used to create a maximum likelihood tree using 1,000 bootstraps and complete deletion of missing data. Otherwise, default settings were used. Molecular weight prediction was executed using ExPasy (https://web.expasy.org/compute_pi/).

### Expression of Recombinant chPTX3

The cDNA sequence of chPTX3 was amplified using full length primers (sense: 5′ TTACAAGGATGACGATGACAAGCTTTCCGTGCTGGATGAAGGC 3′; antisense: 5′ CTGAGATGAGTTTTTGTTCTCTAGATTAAGAAACATACTGAGCTCCTCCATATAG 3′; manufactured by Eurofins Scientific, Hamburg, Germany) and Phusion® DNA polymerase (Thermo Fisher Scientific, Waltham, MA, USA) according to the manufacturers' instructions. As a template, cDNA transcribed from RNA isolated from the spleen of a chicken injected with lipopolysaccharide from *Salmonella enterica* serovar Typhimurium (LPS) (see section Treatment of Chickens With LPS) was used. The obtained sequence was cloned into p3xFlag-Myc-CMV-25 (Sigma-Aldrich, Taufkirchen, Germany) with the NEBuilder Assembly Kit (New England Biolabs, Frankfurt a.M., Germany) according to the manufacturer's instructions. HEK293 cells were stably transfected with the construct p3xFlag-Myc-CMV-25-chPTX3 using X-tremeGENE 9 DNA Transfection Reagent™ (Sigma-Aldrich, Taufkirchen, Germany) according to the manufacturer's instructions. After transfection, 0.25 mg G418 per ml medium was added for clonal selection after every third cell passage. The resulting recombinant protein carries an N-terminal 3x FLAG tag.

### Purification of Recombinant chPTX3

Recombinant chPTX3 was purified from supernatant of stably transfected HEK293 cells using a Glass Econo-Column® (Bio-Rad Laboratories, Munich, Germany) packed with ANTI-FLAG® M2 Affinity Gel (Sigma-Aldrich, Taufkirchen, Germany) and eluted from the gel matrix with 0.1 M glycin-HCl pH 3.5. The pH of the purified protein was neutralized by addition of 30 μl 1 M tris-HCl pH 8.0 per ml eluate.

### Western Blot

After SDS-polyacrylamide gel electrophoresis, Western blot analysis was carried out as described elsewhere (39). Purified recombinant chPTX3 protein was visualized by anti-Flag immunostaining (mouseIgG1 anti-Flag (M2)-HRP; Sigma-Aldrich, St. Louis, MO, USA) and subsequent chemiluminescence. Reducing conditions were established by addition of a final concentration of 25 mM Dithiothreitol (Applichem, Darmstadt, Germany) to the samples before electrophoresis. Deglycosylation was carried out by incubation with PNGaseF (New England Biolabs, Frankfurt a.M., Germany), according to the manufacturer's protocol.

### Analysis of Publicly Available RNA-Seq Data

The chPTX3 nucleotide sequence was used as a query in BLAST searches against Short Read Archive (SRA) files obtained from NCBI (https://www.ncbi.nlm.nih.gov/sra/) using an expected threshold of 1e-10, a word size of 7 and the highest possible number for maximum target sequences. The number of hits was put at a ratio to query size and file size, resulting in a calculated estimate of gene expression (reads per kilobase per million mapped reads; RPKM).

### Chickens

#### Treatment of Chickens With LPS

Six-week-old Lohmann Selected Leghorn chickens (Gut Heinrichsruh, Berglern, Germany), vaccinated only against Marek's disease, were conventionally housed for 5 days prior to the experiment. Water and a commercial diet were provided *ad libitum*. On the day of the experiment, chickens were randomly divided into two groups of six, of which one group was injected intravenously with 10 μg LPS from *S*. Typhimurium (Sigma-Aldrich, Taufkirchen, Germany; L6511) per kg body weight in 100 μl PBS (LPS) while the other group received PBS only (CTR). Three hours after injection, all animals were euthanized and tissue samples were collected. Animal experiments were approved by the Government of Upper Bavaria, License number 55.2-1-54-2531-121-09.

#### Experimental Infection With APEC O1

Chickens of the inbred line 7_2_ (B^2^) were hatched and reared under specified pathogen-free conditions with *ad libitum* access to feed and water. At the age of 2 weeks, the birds were inoculated with 7.3 × 10^4^ colony forming units (CFU), 1.1 × 10^6^ CFU or 8.8 × 10^6^ CFU APEC serotype O1:K1:H7 strain O1 (40) (gratefully received from Prof Lisa Nolan, Iowa State University, USA) in 100 μl PBS or with 100 μl PBS as control administered into the right thoracic air sac. APEC O1 inoculated birds (*N* = 6 per inoculation dose) and control birds (*N* = 3) were culled at 14 hours post infection (hpi), 3 days post infection (dpi) or 7 dpi. All birds were housed in premises licensed under UK Home Office Establishment Licenses (X212DDDBD and XA40CEF03) in full compliance with the requirements of the Animals (Scientific Procedures) Act 1986. Procedures were conducted under license PPL 60/4420 with the consent of The Moredun Research Institute Ethical Review Committee.

### Tissue Sampling

Tissue samples used for this study were collected immediately after euthanasia of the animals and either kept in ice cold sterile PBS for immediate RNA preparation or transferred to autoclaved 1.5 ml snap-cap cups and snap frozen in liquid nitrogen.

### Enumeration of Viable Bacteria

Spleen samples were collected in tubes with 500 μl sterile PBS and weighed. The tissues were homogenized using a TissueLyser II (Qiagen, Manchester, UK), ten-fold serial dilutions in PBS prepared and 100 μl plated onto antibiotic free MacConkey agar plates (Oxoid, Basingstoke, UK). The plates were incubated at 37°C overnight before *E. coli* colonies were counted to calculate the number of colony-forming units (CFU)/g of tissue.

### RNA Preparation

RNA of chicken tissue samples was isolated with TriFast reagent (Peqlab, Erlangen, Germany) according to the manufacturer's instructions. RNA quality was determined using a Bioanalyzer (Agilent, Santa Clara, CA, USA); only RNA with a minimum RNA integrity number (RIN) of 8.0 was used for further analyses.

### cDNA Synthesis

A total of 1 μg RNA per sample was digested by DNAse I (Thermo Fisher Scientific, Waltham, MA, USA) for 30 min at 37°C. DNAse I was inactivated by addition of 2.5 mM EDTA and incubation at 65°C for 10 min. 400 ng of the DNA-free RNA was subsequently transcribed into cDNA using the GOScript kit (Promega, Madison, WI, USA) according to the manufacturer's protocol.

### PCR

PCR analysis was performed using Taq-Polymerase (Solis BioDyne, Tartu, Estonia) and respective buffers according to the manufacturer's instructions, and cDNA of the samples of interest as a template. For analysis of chPTX3 expression in lymphoid tissues, primers spanning the adjoining region of the pentraxin domain at the C-terminus and the N-terminal domain were used to avoid potential amplification of short pentraxins (sense: 5′ ACCAACCTGAGGATGCTG 3′; antisense: 5′ CCGTAGGAGAAGATGATGGTT 3′) at an annealing temperature of 49°C and an extension time of 30 sec. GAPDH expression (Primer sense: 5′ CACGGACACTTCAAGGGCACTG 3′; primer antisense: 5′ CTCCACAATGCCAAAGTTGTC 3′; annealing temperature: 50°C; extension time: 20 sec) was used for normalization. After 34 cycles, PCR products were mixed with one sixth volume of 6x loading buffer with GelRed (GenScript Biotech, Piscataway, NJ, USA) and loaded on a 1.2% agarose gel in TBE buffer (90 mM tris, 90 mM boric acid, 2 mM EDTA-Na_2_, pH 8.0). After voltage application of 130 V for 25 min, resulting bands were visualized under ultravoilet light.

### RNA-seq Analysis

mRNA libraries were prepared from 1 ng RNA from five LPS-treated spleens and five spleens of mock-treated chickens each, using the Sense mRNA Seq Library Kit V2 (Lexogen, Vienna, Austria) according to the manufacturer's instructions. Next-generation sequencing (NGS) was performed on the Illumina HiSeq 1,500 Platform (Illumina, Munich, Germany), producing single-end 100 bp reads (Gene Center, Laboratory for Functional Genome Analysis, LMU Munich, Germany). Read filtering, *de novo* assembly, and alignment to the galGal4 chicken genome was performed in Galaxy (https://usegalaxy.org/) as described elsewhere (41). FASTA files containing the aligned data were created. Only genes with a minimum 4-fold expression change between groups and an adjusted *p*-value of < 0.001 were included in further analysis. The home-made scripts used for detailed analysis workflow are available upon request.

### Quantitative RT-PCR

SYBR Green-based quantitative RT-PCR (qPCR) was performed with cDNA as a template, using GoTaq qPCR Master Mix (Promega, Madison, WI, USA) according to the manufacturer's instructions with a final concentration of 1x of a chicken PTX3-specific commercial QuantiTect Primer Assay (Qiagen, Hilden, Germany) or 300 nM each of GAPDH primers (Primer sense: 5′ AGGGTGGTGCTAAGCGTGTT 3′; primer antisense: 5′ AAGGGTGCCAGGCAGTTG 3′), respectively. Primer efficiency was determined using serial dilutions of a cDNA mix and was 96% for PTX3 and 93% for GAPDH. In addition to the primers, each 25 μl reaction contained 12.5 μl of the GoTaq qPCR Master Mix, 0.25 μl 100x CXR Reference Dye, 4.25 μl of nuclease-free water, and 5 μl of cDNA (10 ng). The cycling conditions were set to 95°C/2 min, followed by 40 cycles of 95°C/15 s, 59 (GAPDH) or 56°C (PTX3)/30 s, 72°C/30 s. Each assay included relevant non-template controls. A melting curve performed following each run confirmed a single, specific PCR product. Raw data created by a 7,300 Real-Time PCR System® (Applied Biosystems, Foster City, CA, USA) was analyzed using the SDS 7,300 software (Applied Biosystems). GAPDH expression was used for normalization (= dCT) and relative expression was calculated for each group (2^dCT^) (42).

### Statistical Analysis

As all data were distributed normally, a *T*-test was applied for statistical analysis. *P*-values were considered significant ^*^ at *p* ≤ 0.05, ^**^ at *p* ≤ 0.01, and ^***^ at *p* ≤ 0.005. Where error bars are shown, they represent the standard deviation for the respective set of samples.

## Results

### Chicken and Mammalian Pentraxin 3 Are Homologs

**Figure 1:**
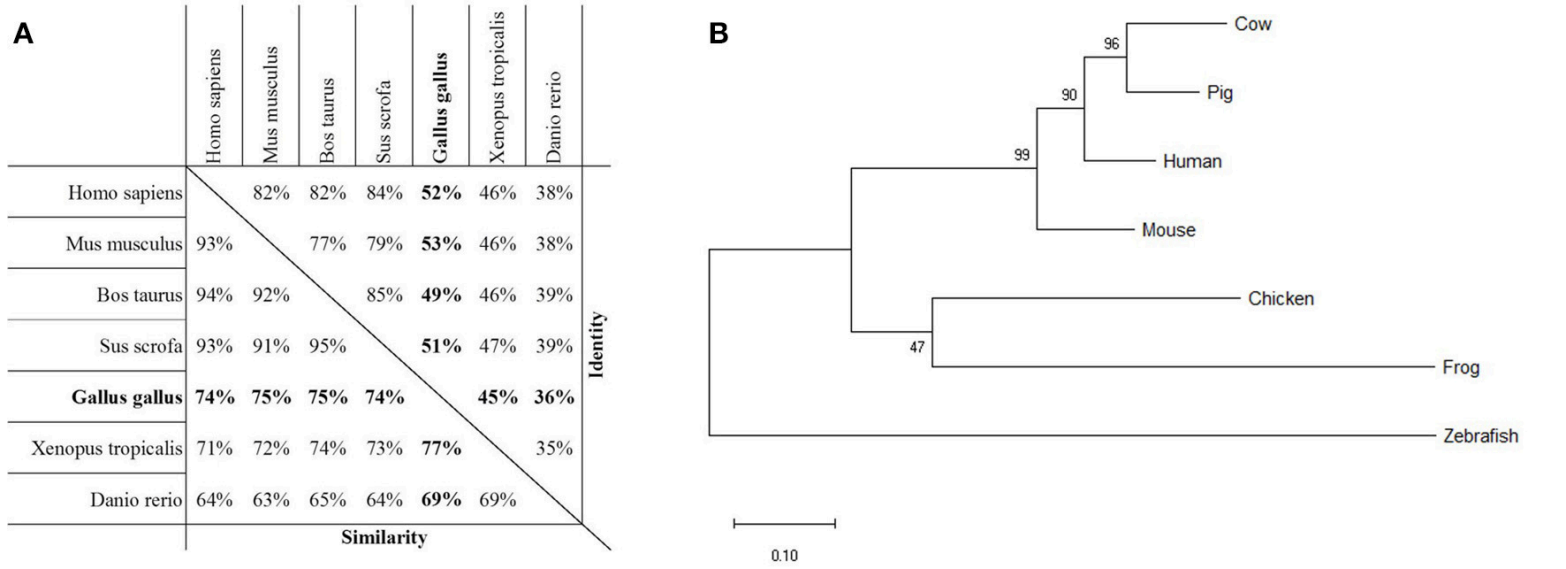
Homology of PTX3 in various species. **(A)** Assessment of protein sequence similarity and identity of PTX3 amino acid sequences of various species, obtained by pairwise alignment. The highest degree of similarity is observed among PTX3 sequences of mammals. The *Gallus gallus* PTX3 sequence (bold) is highly similar to PTX3 of all examined species. **(B)** Phylogenetic analysis by Maximum Likelihood method. The tree with the highest log likelihood (−3,880.21) is shown. The percentage of trees in which the associated taxa clustered together is shown next to the branches. The tree is drawn to scale, with branch lengths measured in the number of substitutions per site.

As a first step toward the characterization of PTX3 in chickens, we evaluated the extent of similarity between the PTX3 protein sequences of chickens and other species. Thereby, we aimed to acquire information on possible alterations in structure of the proteins. The amino acid sequence of chPTX3 was aligned with its counterparts in humans (*Homo sapiens*), mice (*Mus musculus*), cattle (*Bos taurus*), swine (*Sus scrofa*), frogs (*Xenopus tropicalis*), and zebra fish (*Danio rerio*). The alignment (Figure S1) shows an extensive consensus in the pentraxin domain and parts of the N-terminal domain, with an overall sequence similarity of between 69 and 77 %, depending on the species, over the 433 amino acids of the chPTX3 sequence. Overall, 36 to 53 % of the amino acid residues were identical (Figure 1A), verifying that chPTX3 is a close homolog of PTX3 in other species. A phylogenetic analysis of PTX3 protein sequences of the above mentioned species emphasizes the homology and the higher degree of similarity between the mammalian sequences as compared to the lower vertebrates (Figure 1B), as seen in Figure 1A. A closer look at the alignment shows two extended areas within the N-terminal domain in non-mammalian species, which elongate these sequences by a total of roughly 50 amino acids compared to humans and other mammals. The chicken protein features insertions of 27 amino acids between A100 and R101 and 18 amino acids between R188 and A189 of the human protein, respectively (underlined sequence parts in Figure S2). The existence of these elongated sections in the chicken sequence was verified by sequencing of thymus and spleen PCR products spanning this area (Figure S2).

Analogous to the high overall sequence similarity, certain characteristic features are similar between species. For example, the eight amino acid pentraxin signature sequence (dotted line) and a number of disulfide bond forming cysteine residues (triangles) are conserved (Figure S1). Interestingly, cysteine residue C103 in human PTX3 (upside-down triangle in Figure S1) is not conserved in chickens, while all other cysteines known to be structurally important, are. N-glycosylation sites differ more widely between species. Whereas human, mouse, and bovine PTX3 only have one predicted N-glycosylation site each, and porcine PTX3 lacks these sites completely, the non-mammalian species examined here all have two asparagine residues with presumed N-glycosylation (compare asterisks in Figure S1).

### Recombinant chPTX3 Is a Polymeric N-glycosylated Protein

**Figure 2:**
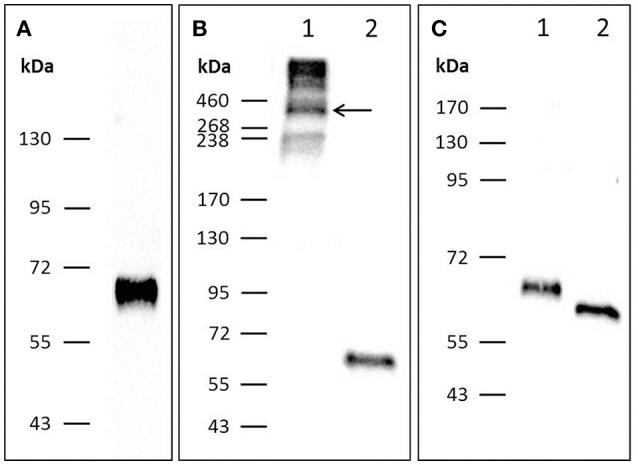
Western blot analysis of PTX3-Flag resolved by SDS-PAGE indicates that chPTX3 has a polymeric N-glycosylated structure. **(A)** Purified chPTX3-Flag from cell culture supernatant under reducing conditions; shows successful expression of the recombinant protein. **(B)** PTX3-Flag under non-reducing (lane B1) and reducing (lane B2) conditions. ChPTX3 is constituted as a polymer. **(C)** Reduced chPTX3-Flag before (lane C1) and after (lane C2) N-deglycosylation. ChPTX3 contains N-glycosylated residues.

To further analyze the structure of chPTX3, its sequence was cloned into p3xFlag-Myc-CMV-25 and transfected into HEK-293 cells to achieve expression of Flag-tagged chPTX3 (Figure 2A). Western blot analysis under non-reducing (Figure 2B, lane 1) and reducing conditions (Figure 2B, lane 2) revealed a polymeric structure of PTX3 in chicken, as reported for mammalian PTX3 (43). The molecular weight (MW) of polymeric PTX3 could not be determined exactly by means of Western blot analysis. However, concluding from Figure 2B, lane 1, the protein is likely to display a variable polymeric organization. The predicted molecular weight of the monomer is 47353.41 Da. As Figure S1 shows, there are two predicted N-glycosylation sites in chPTX3. Western blot analysis of chPTX3-Flag has also been carried out after PNGase F treatment, which eliminates N-glycosylations (Figure 2C). A drop of the MW of monomeric PTX3 after N-deglycosylation of ~5 kDa was detected (Figure 2C, lane 2), which indicates that at least one N-glycosylation site is present within the protein.

### Constitutive PTX3 Expression Differs Widely Between Chicken Tissues

**Figure 3:**
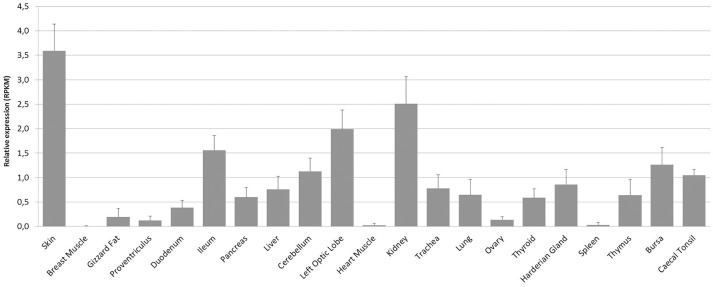
Mean constitutive PTX3 expression in chicken tissue samples from RNA-seq dataset PRJEB12891. RNA-seq data was downloaded from the Short Read Archieve (SRA) database for a total of 168 samples (21 tissues per bird) and blasted for PTX3 reads. Relative expression of PTX3 was assessed for each sample. Data shown represent the mean value of the respective tissue; error bars indicate standard deviation from the mean. ChPTX3 expression pattern varies prominently between tissues. The highest expression is observed in skin and kidney, the least in muscle, ovary, and spleen. Lymphoid tissue samples, except for spleen, express moderate amounts of PTX3 in chickens. *N* = 8.

**Figure 4:**
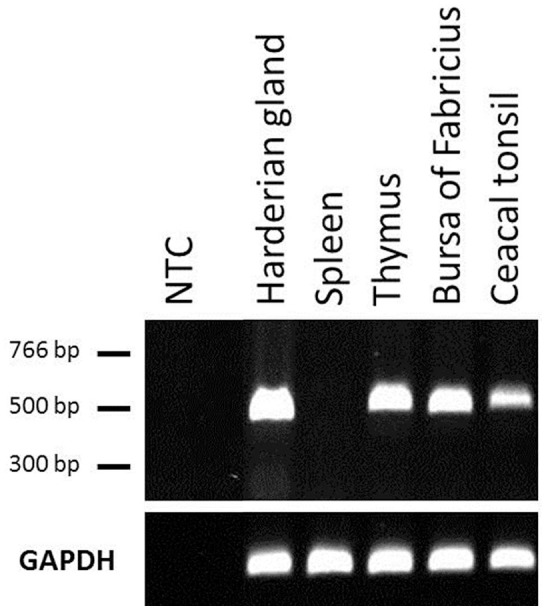
Constitutive PTX3 expression in chicken lymphoid tissue samples, analyzed by RT-PCR. RT-PCR analysis was used to amplify PTX3 transcripts in various tissues. UV-visualization of a subsequent agarose gel electrophoresis is shown. The **(top)** represents PTX3, the **(bottom)** shows GAPDH expression in the same samples. RT-PCR underscores RNA-seq data analysis of chicken lymphoid tissues. NTC, Non-template control.

We next examined the constitutive expression pattern of PTX3 in various chicken tissues. A comprehensive study, executed by The Roslin Institute in Edinburgh, UK, produced RNA-seq libraries of 21 organ samples derived from eight 16 to 17 weeks old healthy female J-line individuals each (NCBI BioProject accession number: PRJEB12891). We found PTX3 transcripts within these data. Figure 3 shows that PTX3 expression varies prominently between native tissue samples. Muscular organs like breast muscle, proventriculus, and heart show a very low expression or no detectable expression at all. In duodenum and ileum as well as in the glands appending to the intestine (pancreas and liver) a moderate PTX3 expression is observed. This is consistent with the PTX3 expression in other mesenchymal, non-lymphoid organs examined (cerebellum, optic lobe, kidney, trachea, lung, ovary, and thyroid), which also express PTX3 to a moderate to high extent. The highest expression was observed in skin samples. Interestingly, all lymphoid organs except for the spleen, which apparently did not express PTX3 in any of the eight examined samples, show moderate PTX3 expression levels, as well. To verify these results, we conducted an RT-PCR analysis on samples of lymphoid tissues of one chicken from our facility. As RNA-seq data had suggested, no expression was found in splenic tissue, while the Harderian gland, thymus, bursa, and caecal tonsil did constitutively express considerable amounts of chPTX3 (Figure 4).

### Bioinformatics and Experimental Approaches Prove Up-Regulation of chPTX3 in Response to Viral and Bacterial Stimuli

To further investigate the importance of chPTX3 as an acute phase protein in chicken, we first analyzed a number of RNA-seq data sets, generated within three different studies in laboratories at the Henan Institute for Science and Technology, the Iowa State University, and the Key Laboratory of Animal Immunology of the Chinese Ministry of Agriculture to gain a comprehensive overview. As Table 1 shows, PTX3 was up-regulated in all three studies. At 1 and 3 dpi with Infectious Bursal Disease Virus (IBDV), PTX3 was 12 and 20-fold up-regulated, respectively, in samples from the bursa of Fabricius. The same tissue up-regulated PTX3 after experimental APEC O1 infection. Although up-regulation could already be shown at 1 dpi, it only became significant at 5 dpi. Another interesting example for a late occurring regulation is the infection with the Gallid Alphaherpesvirus 2 (GaHV2). In this case, PTX3 expression in spleens was slightly elevated at 7 and 14 dpi, but not at 3 dpi, before it finally rose to a 29-fold increase at 21 dpi. The data obtained from these studies suggest that PTX3 may play an important role in experimentally induced inflammatory disease in chickens.

**Table 1:** PTX3 up-regulation as published in RNA-seq datasets.

**Inflamatory stimulus**	**Tissue**	**Study ID**	**Timepoint**	***n***	**Fold change**
IBDV	Bursa	PRJNA369862	1 dpi	3	12
			3 dpi	2	20
APEC	Bursa	PRJNA288323	5 dpi	4	4
GaHV2	Spleen	PRJNA341964	21 dpi	1	29

**Figure 5:**
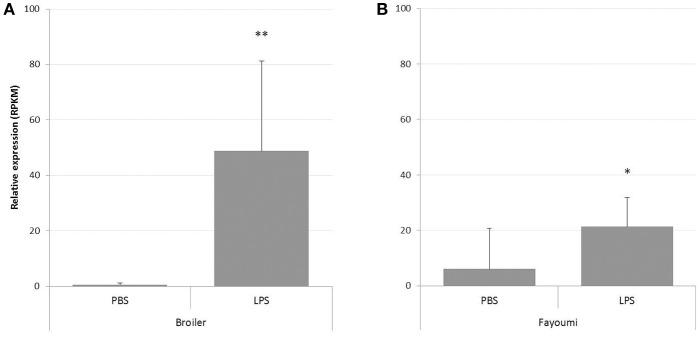
Analysis of RNA-seq data from study PRJNA340891 for PTX3 expression in spleens of LPS-treated chickens. RNA-seq data was downloaded from the Short Read Archieve (SRA) database for a total of 32 samples and blasted for PTX3 reads. Relative expression of PTX3 was assessed for each sample. Data shown represent the mean value of the respective group; error bars indicate standard deviation from the mean. PTX3 expression changes in response to a 3.5 h mock (PBS) or LPS treatment in Broiler **(A)** and Fayoumi chickens **(B)**. LPS enhances PTX3 expression in spleens of Broiler chickens to 95-fold and to 3.5-fold in Fayoumi chickens. ^*^p ≤ 0.05, ^**^p ≤ 0.01. *N* = 8.

Since RNA-seq data had shown a consistent PTX3 up-regulation in a variety of infections, but with distinct regulation patterns, we next analyzed the PTX3 response to a more general inducer of inflammation. We therefore examined data of an RNA-seq study assessing the response in splenic tissue samples of Broiler and Fayoumi chickens to LPS from *Salmonella* Typhimurium (44). Analysis revealed a significant up-regulation in both breeds 3.5 h after LPS treatment compared to mock-treated animals, with 95-fold up-regulation in broilers (Figure 5A) and a 3.5-fold induction in the Fayoumi breed (Figure 5B).

There are only few comprehensive studies on APPs in chickens to date. To gain a thorough picture of the chicken acute phase reaction, we injected LPS intravenously into White Leghorn layer birds and isolated mRNA from spleens 3 h later. An mRNA library of each sample was analyzed by NGS. Processing of NGS data resulted in a total of 175 differentially expressed genes with a fold change of at least four between groups, of which 133 were expressed more intensely in LPS-treated chickens compared to controls (data not shown). The 15 most strongly up-regulated genes are shown in Table 2. Of the 15 most strongly up-regulated genes, 10 (Table 2 No. 3-6, 9-13, 15) are associated with the GO-term “response to stimulus” (GO:0050896), which is twice as many genes as would have been expected statistically. Among the genes associated with GO:0050896, transcription of the acute phase protein PTX3 (Table 2 No. 3) was up-regulated most intensively with a fold-change of 108.

**Table 2:** Fifteen most strongly up-regulated genes in chicken spleens in response to LPS treatment as determined by RNA-seq analysis.

**No**.	**Gene name**	**Gene description**	**Ensembl gene ID**	**Fold change**
1	IL4I1	Interleukin 4 induced 1	ENSGALG00000000081	227
2	AVD	Avidin	ENSGALG00000023622	125
3	PTX3	Pentraxin 3, long	ENSGALG00000028284	108
4	ATF3	Activating transcription factor 3	ENSGALG00000025887	86
5	TNFRSF6B	Tumor necrosis factor receptor superfamily, member 6b	ENSGALG00000006106	79
6	ANGPTL4	Angiopoietin like 4	ENSGALG00000000619	71
7	ARSI	Arylsulfatase family, member I	ENSGALG00000005616	65
8	ST6GALNAC2	ST6 (alpha-N-acetyl-neuraminyl-2,3-beta-galactosyl-1,3)-N-acetylgalactosaminide alpha-2,6-sialyltransferase 2	ENSGALG00000001883	60
9	LHX1	LIM homeobox 1	ENSGALG00000005409	56
10	CA9	Carbonic anhydrase IX	ENSGALG00000021340	54
11	SLCO4C1	Solute carrier organic anion transporter family member 4C1	ENSGALG00000026768	32
12	GCH1	GTP cyclohydrolase 1	ENSGALG00000012200	23
13	IL13RA2	Interleukin 13 receptor, alpha 2	ENSGALG00000020316	19
14	GVIN1	GTPase, very large interferon inducible 1	ENSGALG00000016556	18
15	OASL	2′-5′-Oligoadenylate synthetase-like	ENSGALG00000013723	18

*Shading indicates genes associated with GO term “response to stimulus”(GO:0050896)*.

**Figure 6:**
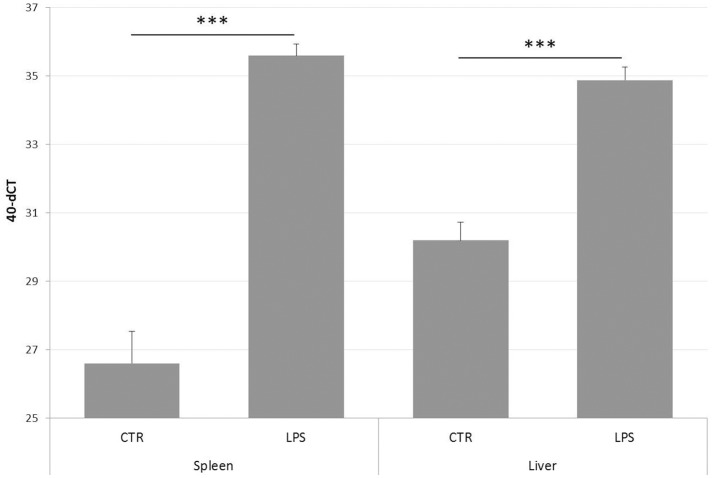
PTX3 expression in chicken spleen and liver after LPS treatment, as determined by qRT-PCR. Spleens and livers of a total of 12 chickens, of which 6 had received LPS intravenously 3 h before scarification, were subjected to RNA extraction. cDNA samples were analyzed using qRT-PCR. Data shown represent the mean value of the respective group; error bars indicate standard deviation from the mean. Mean 40-dCT in control (CTR) spleens is 26.6, and 35.6 in spleens of LPS-treated chickens (LPS). CTR livers display a 40-dCT of 30.2, LPS livers of 34.9. These numbers convert to an expression fold change of 508 for spleens and 26 for livers. The induction of chPTX3 in the LPS group is highly significant (^***^p ≤ 0.005) for both tissues. *N* = 6.

Since NGS data confirmed PTX3 to be strongly induced by LPS in layer chickens, tissue samples of the same experiment were subjected to qPCR analysis. The results are presented in Figure 6 and confirm the RNA-seq findings for the spleens and also show an up-regulation of chPTX3 in the livers of these animals. The difference in normalized cut-off thresholds between LPS-stimulated and control spleens and livers was 9.0 and 4.7, respectively, which converts into fold changes of 507 in spleens and 26 in livers.

**Figure 7:**
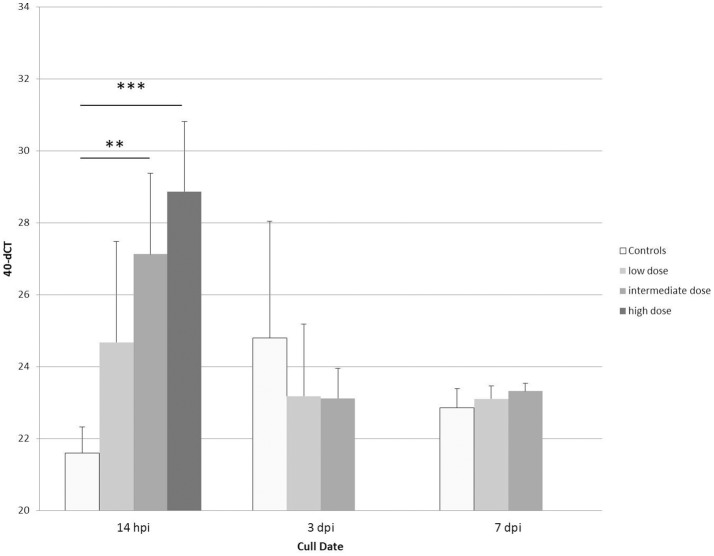
Expression changes of PTX3 in spleens of line 7_2_ chickens at 14 hpi, 3 dpi, and 7 dpi with low, intermediate or high doses of APEC O1, or mock injection, determined by qRT-PCR. Mean 40-dCT values for controls (white bars) and low (7.3 × 10^4^ CFU; light gray bars), intermediate (1.1 × 10^6^ CFU; medium gray bars), and high (8.8 × 10^6^ CFU; dark gray bars) doses were 21.6, 24.7, 27.1, and 28.9, respectively, at 14 hpi. These numbers depict a non-significant expression fold change of 9 between control and low, a very significant fold change of 46 for intermediate (^**^p ≤ 0.01), and a highly significant fold change of 154 for high dose APEC O1 (^***^p ≤ 0.005). Expression changes for APEC-infected birds relative to controls at 3 dpi and 7 dpi were not significant. ChPTX3 expression changes in a dose-dependent manner at 14 h post infection. Data shown represent the mean value of the respective group; error bars indicate standard deviation from the mean. *N* = 3 PBS controls, *N* = 6 APEC O1 low and intermediate dose inoculated birds, *N* = 5 APEC O1 high dose inoculated birds.

**Figure 8:**
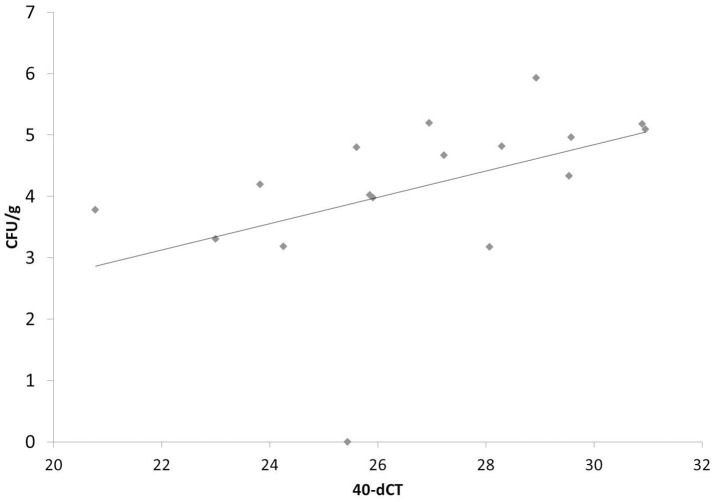
Correlation of PTX3 expression and number of viable bacteria in chicken spleens at 14 hpi after low, intermediate and high dose APEC O1 infection. PTX3 expression correlates significantly (*p* = 0.0313) with the amount of viable bacteria inoculated in the tissue. *N* = 17. CFU/g, colony forming units per gram of splenic tissue.

To further validate our finding that bacterial components, shown here for LPS (Table 2 and Figure 6), are able to strongly induce PTX3 expression in chickens, the next step was to use an experimental infection with APEC O1 and evaluate the PTX3 expression in spleens of infected and control chickens. We therefore selected White Leghorn line 7_2_ chickens, an inbred chicken line reported to be relatively resistant to coinfection with infectious bronchitis virus and APEC infection (45), and injected a total of 51 birds with one of three different doses of APEC O1 or with PBS as a control, respectively. Six infected birds and three of the control group were euthanized at 14 h (hpi), 3, and 7 days post infection (dpi) and spleen samples were collected. Figure 7 shows, that PTX3 expression undergoes a dose-dependent up-regulation at 14 hpi in these chickens. Challenge with the low dose non-significantly amplifies PTX3 expression 9-fold, while significant up-regulation is observed after intermediate dose (46-fold) and high dose (154-fold) APEC O1 challenge. As Figure 8 shows, PTX3 expression correlates significantly with bacterial load numbers in the spleen. In agreement with the low numbers of viable bacteria recovered from the spleen at 3 and 7 dpi (data not shown) there is no significant difference in PTX3 expression between control and infected birds at these time points, which underlines the importance of chPTX3 as a marker for the acute phase of infection.

## Discussion

The role of PTX3 in the innate immune system, where it acts as a pattern recognition molecule and potent complement activator, and the rapid change of its expression in the course of the acute phase of infections have been studied intensely in mice and men (2, 32, 46, 47). However, the expression, structure and function of chicken PTX3 has previously been neglected. Our recent microarray studies connected PTX3 to the acute phase reaction in chickens for the first time (38). However, its annotation in the chicken genome remained to be verified.

To address this question, we first examined the protein encoded by the gene annotated as chPTX3 to assess the sequence similarity to PTX3 of other vertebrates. With identical amino acids representing 36 to 53% of the sequences and an overall similarity of up to 77% with minor species-specific variations, the chPTX3 sequence proved to be highly similar to other PTX3 sequences. The pentraxin signature sequence is an eight amino acid motif within the C-terminal so-called pentraxin domain and is present in all members of the pentraxin family (32). It is also conserved in chickens. Interestingly, mammalian PTX3 sequences are more similar to chicken PTX3 than are those of lower vertebrates like *Xenopus tropicalis* and *Danio rerio*. Closer examination of the sequences nonetheless shows that there are some distinct features between mammals and non-mammalian species examined here. One notable difference is the deletion of significant parts of the sequence in the course of mammalian evolution. These might influence the quaternary structure and thereby the function of chPTX3.

Human PTX3 has previously been shown to form asymmetric, cyclic octamers (48) and cysteine residues are of major importance to the formation of their critical intra- and interchain disulfide bonds (43). By aligning PTX3 amino acid sequences of human, mouse, cow, pig, chicken, frog, and zebra fish, we found that most of the cysteine residues, which are in humans and mice contributing to the formation of the polymeric structure of PTX3 (43, 48), are conserved in chickens. Contrarily, one of them, C103 in human PTX3, is not. C103 has been shown to confer the organization of PTX3 protomers into either dimers or tetramers, which then form the final octamer (48). Western blot analysis of recombinant chPTX3 reveals a polymeric structure with a prominent MW drop to ~65 kDa after disulfide bond reduction and thereby proves its polymeric structure. The polymer's exact MW and thereby its structural organization, however, remains to be determined. These findings indicate a major divergence in quaternary structure between mammalian and chicken PTX3.

Protein glycosylation was shown to be of significant functional relevance in human PTX3. The terminal glycan of the PTX3 N-glycosylation site at N220 is sialic acid. This molecule is one of the main effectors of the binding characteristics of PTX3 to pathogens and mediators of immunological functions (46, 49). The sialic acid residue of N220 obscures ligand binding sites on the protein surface of human PTX3, represented by residues K214, E252, K255, and R332. Preventing excessive C1q binding, PTX3 sialylation thus mediates down-regulation of complement activation (46). The complement system is activated in a highly potent manner only by deglycosylated PTX3 (49). In addition, leukocyte trans-endothelial migration is inhibited through occupation of P-selectin, which is essential for the mediation of leukocyte rolling, by sialic acid residues on the surface of human PTX3 (50). In contrast to its immune-enhancing role, deglycosylation of PTX3 during influenza infection abolishes its anti-viral activity. Only an intact sialic acid residue on PTX3 is able to mimic hemagglutinin binding sites on host cells and by that means act as a decoy receptor for the virus (51). PTX3 deglycosylation in this case hence exacerbates infection (35). The same mechanism probably applies to the defense against other viruses with similar infection strategies (52). Our analysis of PTX3 protein sequences revealed that the number of N-glycosylation sites differs widely between species. Like human PTX3, the mouse and bovine proteins also have one predicted N-glycosylated asparagine residue (N-X-S/T, where X can be any amino acid except for P). In addition, two of the four amino acids interacting with the sialic acid residue at N220 in humans are conserved in each of these species. In contrast, pig PTX3 does not have any predicted N-glycosylation sites, which may potentially contribute to their high susceptibility to a variety of influenza virus infections (53). Most interestingly, prediction of N-glycosylation sites for the three non-mammalian species examined here resulted in two hits each, of which one is located within the N-terminal and the C-terminal domain, respectively. Here we prove the presence of at least one N-glycosylation site in chPTX3 by a MW drop of deglycosylated compared to untreated recombinant chPTX3. Due to the fact that the composition of glycan substitutions is very variable, not only between species but also in a tissue-specific manner (49), further analysis of these structures will be necessary to assess whether only one or both of the predicted N-glycosylation sites are indeed glycosylated in chickens and if tissue-specific glycosylation might alter the protein function in this species.

It has been published earlier that most APPs are mainly synthesized and released by hepatocytes (54), but newer studies indicate a more variable origin (55). In consensus with that, mammalian PTX3 is expressed in a variety of cells under inflammatory conditions and has been shown to be constitutively expressed first and foremost in lymphatic endothelial cells (56). We therefore next investigated the chPTX3 expression pattern under non-inflammatory and inflammatory conditions. We first searched public RNA-seq databases for studies on chicken tissues and chose one especially comprehensive study. Interestingly, most analyzed chicken tissues constitutively expressed PTX3. Exceptions were muscular organs (breast muscle, proventriculus, and heart muscle), ovary and spleen. In contrast, skin and kidney showed the most prominent constitutive expression. PTX3 expression has previously been shown to be constitutive in human kidney, as well (57), whereas skin samples have, to the best of our knowledge, not been analyzed for constitutive PTX3 expression in mammals so far. Interestingly, Doni and coworkers recently reported a strong association of PTX3 with the process of wound healing in mice (47). As the skin forms the outer body barrier (58) and the kidney is one of the main detoxification organs (59), these tissues can *in vivo* probably hardly ever be considered “unstimulated” and their strong expression of an inflammation-responsive molecule is no surprise.

Highly surprising, on the other hand, was the finding that the main lymphoid organ of birds (who lack lymph nodes), the spleen, did not considerably express chPTX3 in any of the eight sampled individuals. This finding in chickens is contrary to mammals, where lymphatic endothelia show a prominent constitutive expression of PTX3 (56). The lack of constitutive PTX3 expression in this immunologically highly relevant tissue points to active regulatory mechanisms. As recently shown in human primary cells and cell lines, PTX3 expression is regulated by three upstream regulatory elements, of which one is also highly conserved in chickens. It was shown furthermore, that in colorectal cancer cells, PTX3 is silenced by epigenetic modifications within these regions, that are, among other factors, induced by a high activity of STAT3 in these cells (60). STAT3 or another regulator of immune functions might be responsible for depression of PTX3 in unstimulated chicken spleens to prevent overstimulation of the immune system. Complete elucidation of the non-redundant immunological functions of the avian spleen, however, remains yet to be achieved and might help to explain PTX3 regulation. Other major lymphoid organs in chickens, including the Harderian gland, thymus, bursa of Fabricius and the caecal tonsil, did constitutively express PTX3. This is consistent with the findings in mammalian lymphatic endothelia (56). To verify the RNA-seq data for lymphoid tissues, we performed an RT-PCR analysis of chicken samples from our facility and were able to confirm the RNA-seq results by demonstrating the absence of constitutive chPTX3 expression in spleen, but strong signals in the other four lymphoid tissue samples.

As a next step, we aimed to characterize PTX3 expression changes in response to inflammation. We analyzed RNA-seq data from various studies addressing the acute phase response in chickens and thereby detected a PTX3 up-regulation in a number of highly relevant viral and bacterial diseases, including IBDV, APEC, and GaHV2, the pathogen inducing Marek's Disease.

Analysis of the dataset drawn from an experimental infection with 10^8^ CFU APEC O1 (61) resulted in a PTX3 expression increase of only 4-fold between controls and diseased birds, occurring at 5 dpi. On the other hand, LPS induced a 96-fold and a 3.5-fold expression change for Broiler and Fayoumi chickens, respectively, in another study of which we examined the RNA-seq data (44). This prominent difference in PTX3 induction demanded further investigation of the overall chicken acute phase reaction. Our next approach hence was to perform an RNA-seq analysis on spleen samples taken from White Leghorn Layer chickens from our facility, which had been LPS- or mock-treated for 3 h. As would have been expected after LPS-treatment, the majority of the most strongly up-regulated genes cluster to the GO term “response to stimulus.” Supporting our thesis that PTX3 is an important acute phase protein in chickens, the PTX3 gene was up-regulated 108-fold in LPS-treated chickens and showed up on position 3 of the most highly up-regulated genes. Narrowing the list down to only those genes associated with “response to stimulus,” it even was the most strongly regulated gene. A subsequent qPCR analysis of spleen and liver samples of six LPS- and mock-treated chickens verified the results obtained by NGS. The 15 most strongly down-regulated genes, in contrast, do not clearly cluster to any immune response-associated GO term in particular, but are mostly linked to processes concerned with tissue homeostasis (data not shown). The down-regulation seen here is exemplary for the fact, that under inflammatory conditions, tissue and endothelial integrity are impaired (62, 63). Interestingly, none of the genes encoding proteins that are considered classical APPs in mammals, e.g., CRP and SAP, show up in this analysis even though they have repeatedly been described in birds (19, 64). Nor did we detect any known chicken APPs, like SAA, ceruloplasmin, ovotransferrin or α1-acid glycoprotein, to be differentially regulated in this experiment. These data prove the induction of PTX3 in genetically diverse chickens such as White Leghorn layers, Broilers, and Fayoumi chickens and indicate, that PTX3 might play an important role as a major APP in chickens.

The next step after proving that chicken PTX3 is inducible by an inflammation-inducing but non-infectious agonist *in vivo* was to perform a comprehensive kinetic study using the White Leghorn inbred line 7_2_, which has been shown to be relatively resistant to coinfection with infectious bronchitis virus and APEC (45). We thereby aimed to assess if PTX3 is an appropriate marker of subclinical disease and whether its expression would correlate with the severity of infection. We compared PTX3 expression changes in spleens triggered by three different doses of APEC O1 at three early time points, 14 hpi, 3 dpi and 7 dpi. It is shown here that all infected chickens significantly amplify their PTX3 expression, but only at 14 hpi. The PTX3 expression increases in a challenge dose-dependent manner. Expression fold changes are 9, 46, and 154 for low, intermediate and high dose APEC O1-treatment, respectively. The changes in intermediate and high dose expression, as compared to controls, are of high significance. PTX3 expression in splenic tissue correlates significantly with the bacterial burden in the same tissue samples, which makes PTX3 a valuable indicator of infection severity. Interestingly, PTX3 levels do not differ between controls and infected chickens at the later time points, when bacterial burdens are decreasing toward zero again. The differential PTX3 expression between challenge doses and its correlation with the number of viable bacteria in the respective samples establish chPTX3 as a potential tool for the diagnosis of inflammatory disease and represent indicators of even subclinical pathologic conditions.

In conclusion, the study presented here shows that PTX3 is of major interest concerning the chicken acute phase reaction. We showed that the chPTX3 protein, regardless of a high overall sequence similarity, differs from the well-described mammalian PTX3 by structural aspects, which are very likely to convey differential functional characteristics. Furthermore, we succeeded in expressing recombinant chPTX3, which enables us to generate specific monoclonal antibodies. Detection of the PTX3 protein in serum and other biological samples will thereby be rendered possible. The results presented here build a solid base for further studies on chPTX3 ligands, binding properties, and association to immunological pathways in the future. We furthermore prove here that PTX3 is expressed in a large variety of chicken tissues and that the chicken's main lymphoid organ, the spleen, does not, like the other lymphoid tissues in chickens and mammals, constitutively express PTX3, but rigorously responds to inflammatory stimulation. Inflammation, however, induces a stimulus- and infection dose-dependent PTX3 response in spleen and other tissues on mRNA level. Based upon the first insights on chPTX3 presented here, further studies may provide evidence for its eligibility as a general marker for the acute phase of inflammation and thereby support diagnostics and disease control for a progressive poultry industry.

## Data Availability Statement

NGS data generated for this study can be found in ArrayExpress, hosted by the European Bioinformatics Institute (https://www.ebi.ac.uk/arrayexpress/) under accession number E-MTAB-7082.

## Ethics Statement

The first animal experiment in this study (LPS injection; Germany) was carried out in accordance with the recommendations of the TierSchVersV, adopted by the Bundesministerium für Ernährung, Landwirtschaft und Verbraucherschutz. The protocol was approved by the government of Upper Bavaria, license number 55.2-1-54-2531-121-09.

The second animal experiment (APEC infection; UK) was carried out in accordance with the recommendations of the requirements of the Animals (Scientific Procedures) Act 1986 by the UK Home Office Establishment. The protocol was approved by The Moredun Institute Ethical Review Committee under PPL 60/4420.

## Author Contributions

NB, SR, AS, SH, TC, AA, LV, and BS performed experiments. NB carried out RNA-seq data analyses. SR and SH performed NGS data acquisition and analysis. DE supervised bioinformatics analyses. BK and MS planned the study and analyzed data. NB, AA, and BK wrote the manuscript. All authors contributed to, read and approved the final manuscript.

### Conflict of Interest Statement

The authors declare that the research was conducted in the absence of any commercial or financial relationships that could be construed as a potential conflict of interest.

## Abbreviations

APEC: Avian Pathogenic *E. coli*
APP: acute phase protein
APR: acute phase response
CFU: colony forming units
chPTX3: chicken Pentraxin 3
CRP: C-reactive Protein
CTR: control
dpi: days post infection
GaHV2: Gallid Alphaherpesvirus 2
hpi: hours post infection
IBDV: Infectious Bursal Disease Virus
LPS: lipopolysaccharide from *E. coli*
MBL: mannose-binding lectin
MW: molecular weight
NGS: next generation sequencing
RIN: RNA integrity number
RPKM: reads per kilobase per million mapped reads
SAA: serum amyloid A
SAP: serum amyloid P
SRA: sequence read archive.
