# Supplementary figures and images for: The Long Pentraxin PTX3 Is of Major Importance Among Acute Phase Proteins in Chickens
Source: Front Immunol. 2019 Feb 1;10:124. doi: 10.3389/fimmu.2019.00124 (PMC6367253; doi:10.3389/fimmu.2019.00124)

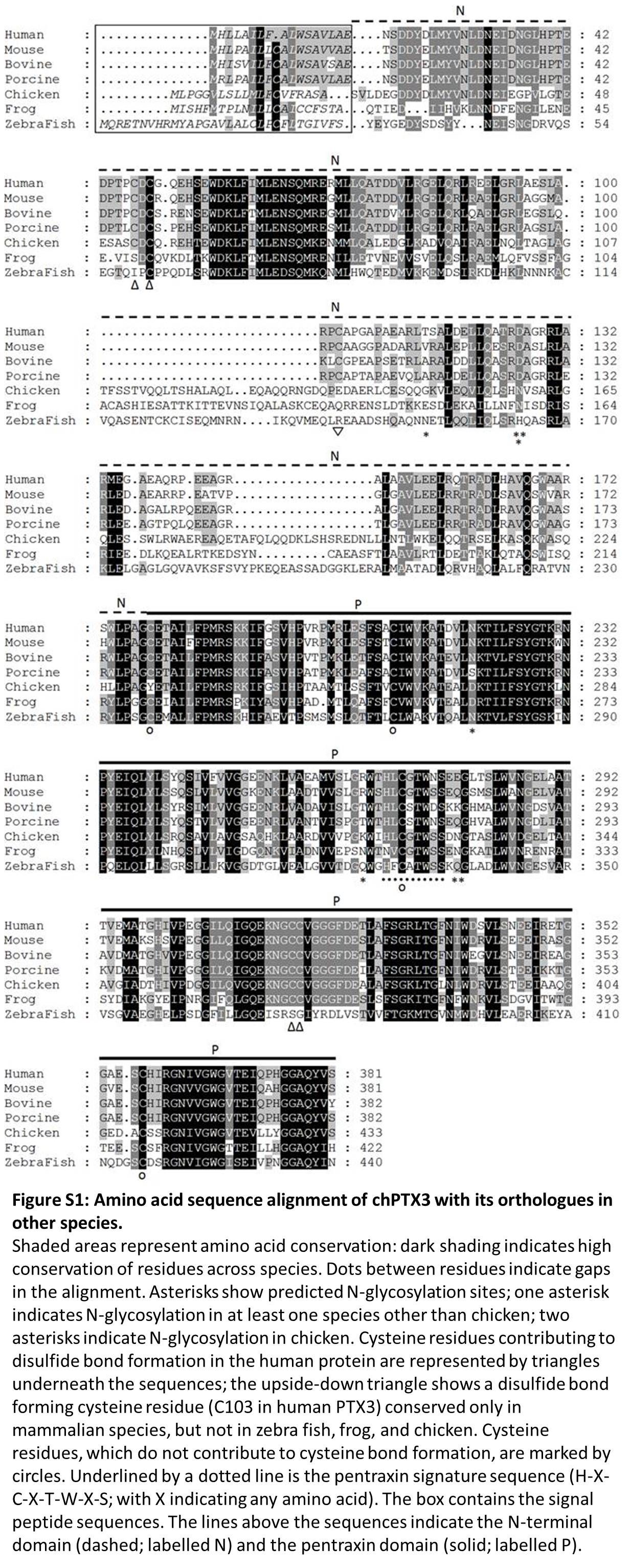

Supplement: Supplementary file 1 [file Image_1.JPEG]

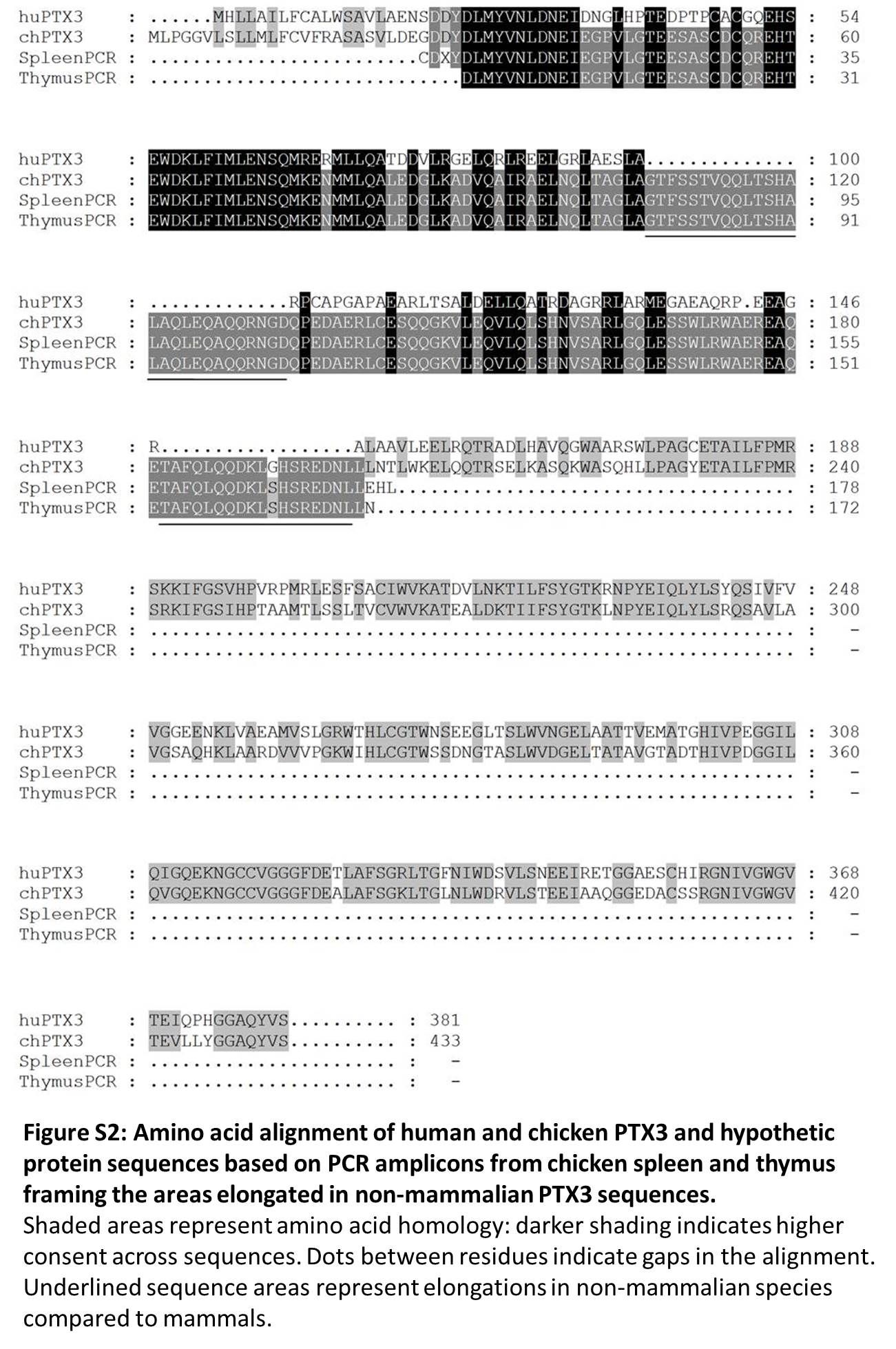

Supplement: Supplementary file 2 [file Image_2.JPEG]
